# Welfare of invertebrates: a pilot study on a new land snail stunning technique

**DOI:** 10.1038/s41598-024-58133-4

**Published:** 2024-04-10

**Authors:** Paola Fossati, Federico M. Stefanini, Giuliano Ravasio, Umberto Coerezza

**Affiliations:** 1 Department of Environmental Science and Policy – ESP, Università degli Studi di Milano, Via G. Celoria, 10, 20133 Milano, Italy; 2 Department of Veterinary Medicine and Animal Sciences, Università degli Studi di Milano, Via Dell’Università 6, 26900 Lodi, Italy; 3 Veterinary Department and Safety of Foods of Animal Origin - ATS Insubria, Varese, Italy

**Keywords:** Zoology, Animal behaviour, Animal physiology, Psychophysics

## Abstract

The almost complete absence of regulations to protect invertebrates is a common condition in legal systems, including the European one, especially when it comes to invertebrates intended for human consumption. Thus, in the vast majority of cases, edible invertebrates do not receive even the most basic protection at slaughter. Despite recent research indicating that invertebrates are capable of feeling pain and stress, the humane step of stunning is not used on them. This is also the case for land snails, which are gastropod invertebrates whose consumption has now reached significant levels, already involving tonnes and that is expected to increase significantly as edible snail farming becomes more popular as a relatively low-cost, easy-to-perform, and sustainable alternative animal husbandry, thereby making land snails an increasingly economically important species. This paper presents and investigates a proposed stunning method based on the immersion of mollusks in CO_2_-supplemented and refrigerated water that could be used in the snail meat production chain to reduce the slaughter suffering of millions of these invertebrates. To this end, body condition descriptors (hemolymph parameters) in snails were determined before and after CO_2_ treatment in cold water, while generating useful data for defining a preliminary set of reference intervals for basal values.

## Introduction

Invertebrates are a group of animal species that, in addition to accounting for the vast majority of those on the planet, are gaining attention for their potential use as food. Land snails, in particular, are proving to be an increasingly appealing breeding species for human food production. Farmers benefit from farming these gastropod mollusks in terms of economics and resource utilization, and it is also liked and valued for the production of meat, which is thought to have good nutritional and organoleptic properties. According to recent research, land snail consumption peaked at 43,000 tonnes in 2016, and this figure is expected to rise by at least 50,000 by 2025 (World: Snails (Except Sea Snails)—Market Report, 2016).

For centuries, the use of land snails in gastronomy has been a part of the culinary tradition in many Mediterranean countries^1^. Land snails, for example, have been present in Italian cuisine since the time of the ancient Romans. Based on the discovery of shells at several archaeological sites^2^, the earliest known breeding of these animals can be traced back to 50 B.C., and even Gaius Pliny the Elder (23–79 A.D.) mentions the edible species of snail in his "Naturalis historia," suggesting a recipe for serving them^3^. Furthermore, it is well known that snail meat is widely consumed in France, Spain, Portugal, Greece, and Morocco^4^. The demand is increasing. It is estimated that production in Italy alone has increased by 325% over the last 20 years, reaching 44 thousand tonnes, including live and preserved product^5^, while FAO data confirm the gradual and steady increase in production worldwide (Parameters: FAO data)^6^. Farmed snails are members of the phylum Mollusca and the class of Terrestrial Gastropods (aquatic species also exist). *Cornu aspersum* (Müller, 1774) and *Helix pomatia* Linnaeus, 1758 are the most prevalent in the Mediterranean and Atlantic areas of Europe. The most common and profitable eliciculture involves the use of snails of the species *Cornu aspersum*, which are very productive animals and easy to raise, even in spaces that are not necessarily large and with little investment other than initial costs^7–9^. Land snails, like other invertebrates, have pain system elements, avoidance reactions to pain stimuli, and opioid-mediated stress responses similar to mammals^10–13^. However, unlike other invertebrates, land snails are almost completely unprotected by animal welfare laws. The lack of legislation is total with regard to edible land snails. In consequence, no industry regulation exists to define the requirements and treatment methods for farmed snails that enter the food chain (Exceptions are cephalopods, which are protected when used in animal research in the European Union, and species listed in the annexes of the Habitats Directive. The Swiss Confederation regulation OPA n 455.1 New Text in accordance with No. I of the O. of January 10, 2018, in force since March 1, 2018 (AS 2018 573) prohibits certain transport and storage practices for decapod crustaceans and forbids immersing them alive and not stunned in boiling water for cooking, requiring them to be stunned before killing; this practise can only be done by qualified personnel. In Italy, some regional laws establish provisions for the protection and conservation of so-called 'small fauna', which includes invertebrates, but only those living in the wild in their natural habitat). This is also true for the slaughter phase, which occurs in both family or small catering consumption as well as industrial realities using the traditional system of boiling live snails without a prior stunning phase inducing unconsciousness with minimal distress and pain. As a result, there is currently no humane way to stun these animals and kill them while they are unconscious. The ideal method should be painless and cause a rapid loss of consciousness. Furthermore, it should be human-safe, not impressive to the eye, inexpensive, easy to perform on both small and large scales, and protect both animal welfare and organoleptic quality and food safety.

This paper proposes a technique for stunning land snails for slaughter that can be easily applied to any number of snails and could improve protection guarantees for the vast majority of these animals used for food production. The study was conducted on *Cornu aspersum* (Müller, 1774), the most widely bred and marketed species, while collecting data on the species’ main hemolymph parameters, which were biochemically studied using a hemogasanalysis instrument. A series of basal values, useful for defining a preliminary set of reference intervals, were identified through Bayesian analysis of the collected data. In addition, an atraumatic method of extracting hemolymph from ground snails was performed.

## Cornu aspersum's biological and anatomical characteristics

**Figure 1:**
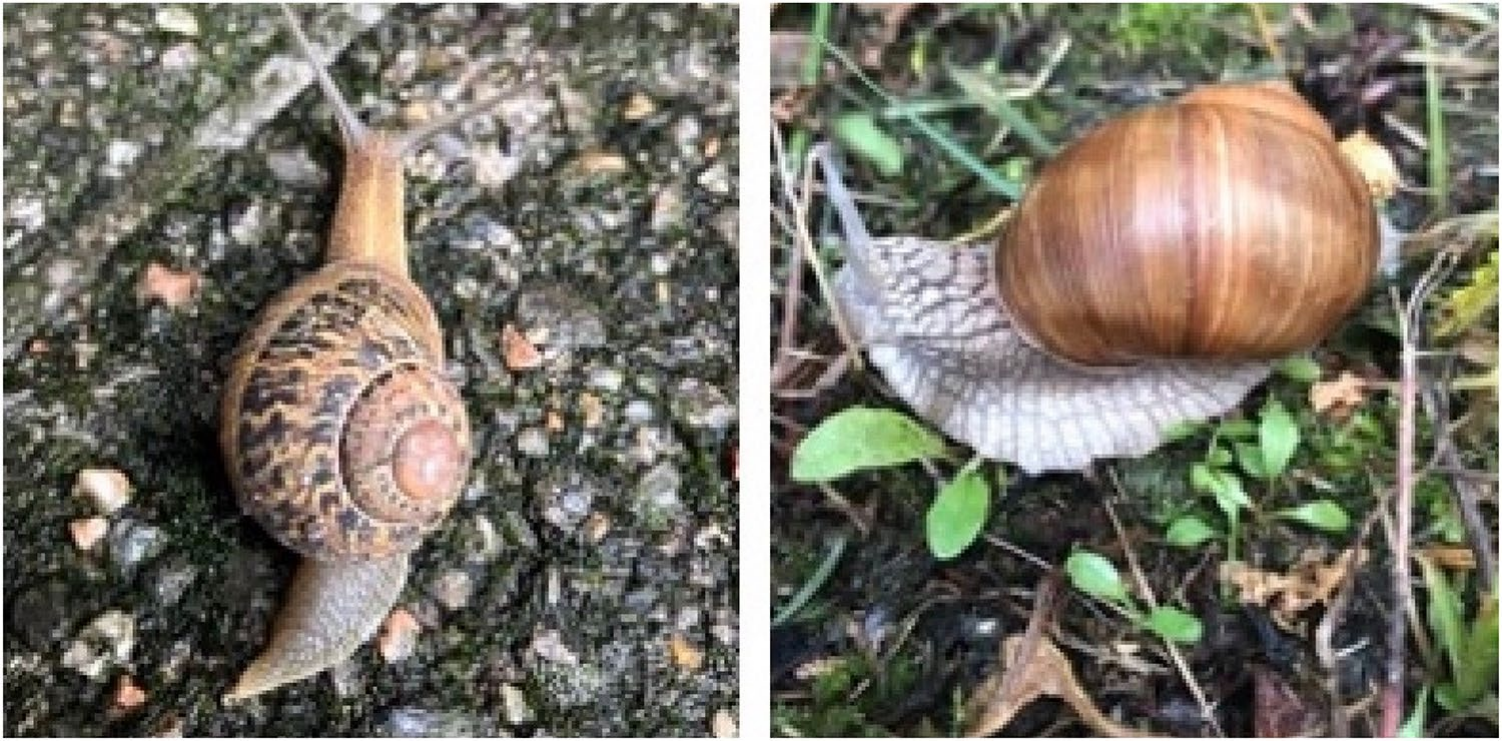
Cornu aspersum.

*Cornu aspersum* (former *Helix aspersa*) is a common snail naturally found throughout the Italian peninsula, particularly in the Mediterranean basin (Fig. 1). It is a terrestrial gastropod mollusk of the Helicidae family. Its shell is cone-shaped, with 3–4 coils developed around a central axis (columella) and can hold the animal’s cephalo-podal mass. In fact, the snail has a ventral muscular foot that allows it to move. The internal organs are contained within the body in a single cavity, which is protected by a mantle that lines the inside of the shell.

Snails are pulmonated animals. Their nervous system is ganglionic, with ganglia of nerve cells interconnected by bundles of nerve fibers. Although they lack a true brain, snails have been observed to have associative thinking skills, implying that this learning ability, while primitive, allows them to experience pleasure and pain (as sentient beings)^14,15^. The evolution of terrestrial gastropods has revealed that they evolved from aquatic forms^16^. The importance of water in terrestrial gastropod survival has been extensively studied^17–19^. Even today, land snails contain a large amount of water and maintain a constant, complete aqueous body cover due to the production of a hygroscopic slime. This condition allows them to withstand more water immersion before drowning than other pulmonated organisms^20^. There are also terrestrial snails’ species that can survive underwater^21^.

### Terrestrial gastropods’ respiratory system

Snails breathe through a valved opening called a pneumostome that connects to a rudimentary lung made up of an evolved lung sac in the mantle cavity. The pneumostome opening regulates both internal CO_2_ pressure and the need to inhale oxygen-rich fresh air. It has been demonstrated that oxygen is introduced into the respiratory surfaces of lung gastropods via water. In fact, an aqueous layer coats both the walls of the lung cavity and the outer surface of the body, allowing skin respiration^22^. Furthermore, the snail lung can adapt to aquatic respiration, albeit with less advantageous gas exchanges than air respiration. The exchange of gases within the organism is based on the phenomenon of diffusion, which is influenced by the difference in partial pressures of each of them between the environment outside and inside the organism itself and, as previously stated, occurs primarily through the pneumostome, the opening of which is stimulated by the lowering of oxygen pressure inside the mollusk as well as by hypercapnia, with a response mediated by CO_2_-sensitive cells present in the central nervous system of the snail^23^. Some research suggests that hypoxia with hypercapnia, as well as subsequent acidification of intracellular and tissue pH, may govern a reversible transition phase between the active and quiescent condition in lung mollusks^24,25^. These findings lend support to the hypothesis that hypercapnia slows metabolism in such animals, allowing them to enter a dormant state. It is a reversible state of quiescence that, in snails, is comparable to stunning. In fact, according to Regulation (EC) No. 1099/2009 on the protection of animals at the time of killing, ‘stunning’ means any intentionally induced process which causes loss of consciousness and sensibility without pain, including any process resulting in instantaneous death (Art. 2, letter f). Contrary to what generally happens in vertebrate species when using the stunning methods listed in Regulation (EC) No. 1099/2009, snails are able to recover after the treatment described^26^.

## The legislation governing the slaughter of invertebrates

Existing general requirements for their killing and related operations require that animals be spared avoidable pain, distress, or suffering (Regulation No. 1099/2009/EC, Art. 3). To that end, stunning before killing has been made mandatory. This practice, however, does not apply to the slaughter of invertebrates, which are exempt from the application of Regulation No. 1099/2009/EC, establishing criteria for the protection of animals used for food production that are limited to vertebrate animals.

In the absence of current regulations requiring prior stunning, land snails produced by heliciculture and intended for gastronomic consumption are slaughtered directly with the traditional boiling of live and conscious mollusks.

## Results

### Summary statistics

In Table 1, summary statistics are shown for each variable given the selected treatment after removing extreme observations (outliers). In Table 1S of the Supplementary Material, summary statistics are also calculated but using raw data, that is without removing extreme observations (outliers). Box plots based on the quantiles shown in Table 1 are also included in the Supplementary Material.

**Table 1:** Summary statistics given treatment (outliers removed).

Variable	Treatment	Min	Q.25	Mean	Q.50	Sd	Q.75	Max	n_obs
pH	Bas	7.37	7.51	7.53	7.54	0.05	7.57	7.62	73
pH	CO_2_	6.54	7.06	7.15	7.21	0.21	7.26	7.55	31
pCO_2_	Bas	15.60	21.25	23.77	23.40	4.12	25.75	38.30	67
pCO_2_	CO_2_	46.20	84.07	129.53	121.65	57.14	159.42	260.00	28
log10pCO_2_	Bas	0.56	0.98	1.07	1.06	0.18	1.15	1.62	70
log10pCO_2_	CO_2_	1.53	1.87	2.04	2.05	0.25	2.17	2.58	29
pO_2_	Bas	32.50	63.70	92.00	93.60	35.26	119.10	163.20	73
pO_2_	CO_2_	20.70	40.10	63.97	50.00	35.07	78.15	165.90	31
Na_p	Bas	60.10	72.17	76.95	75.95	7.68	80.90	94.20	30
Na_p	CO_2_	58.10	67.60	71.85	72.20	6.15	75.50	85.60	23
K_p	Bas	1.83	2.02	2.36	2.33	0.50	2.54	4.10	31
K_p	CO_2_	1.98	2.17	2.50	2.32	0.53	2.67	4.03	21
Cl_m	Bas	61.30	70.53	74.03	74.10	6.01	76.83	88.40	30
Cl_m	CO_2_	56.90	63.00	67.27	67.20	5.91	68.50	78.80	23
Ca_pp	Bas	2.73	3.41	3.73	3.74	0.54	4.12	4.72	31
Ca_pp	CO_2_	3.47	4.90	6.29	6.28	1.98	7.22	10.97	23
TCO_2_	Bas	13.40	19.55	21.07	21.30	3.03	22.63	27.80	60
TCO_2_	CO_2_	13.80	37.80	49.05	49.60	17.28	61.15	99.10	27
nCa	Bas	2.97	3.71	4.01	3.98	0.55	4.37	4.95	30
nCa	CO_2_	3.64	4.15	5.38	5.25	1.44	6.26	8.34	20
pH (TC)	Bas	7.37	7.63	7.72	7.75	0.11	7.81	7.87	73
pH (TC)	CO_2_	6.69	7.22	7.28	7.28	0.20	7.41	7.65	31
pCO_2_ (TC)	Bas	7.40	11.05	15.46	13.00	6.09	19.50	37.20	71
pCO_2_ (TC)	CO_2_	22.00	56.40	87.05	75.30	42.61	113.90	184.90	29
pO_2_ (TC)	Bas	9.80	23.10	54.38	44.70	37.56	79.40	159.70	73
pO_2_ (TC)	CO_2_	6.20	13.72	41.85	26.25	38.78	63.40	165.90	30
SBC	Bas	18.40	22.75	24.55	24.50	3.03	26.28	35.50	70
SBC	CO_2_	5.10	27.68	33.67	33.50	11.41	40.08	53.90	26
HCO_3__m	Bas	13.00	18.80	20.38	20.35	3.13	21.83	30.80	68
HCO_3__m	CO_2_	10.20	32.95	44.55	45.60	16.16	54.70	91.20	27
A	Bas	101.90	124.67	133.55	137.15	11.38	139.98	179.30	72
A	CO_2_	5.70	40.20	67.58	63.50	37.39	92.95	134.70	24
Osm	Bas	125.10	145.40	153.92	151.95	13.75	161.93	186.80	28
Osm	CO_2_	119.90	138.20	145.42	146.25	11.94	153.07	171.00	20

### Inferring intervals of commonly observable values for pretreatment variables

The procedure to estimate endpoints of intervals describing common observable values is summarized below for the pH variable.

**Figure 2:**
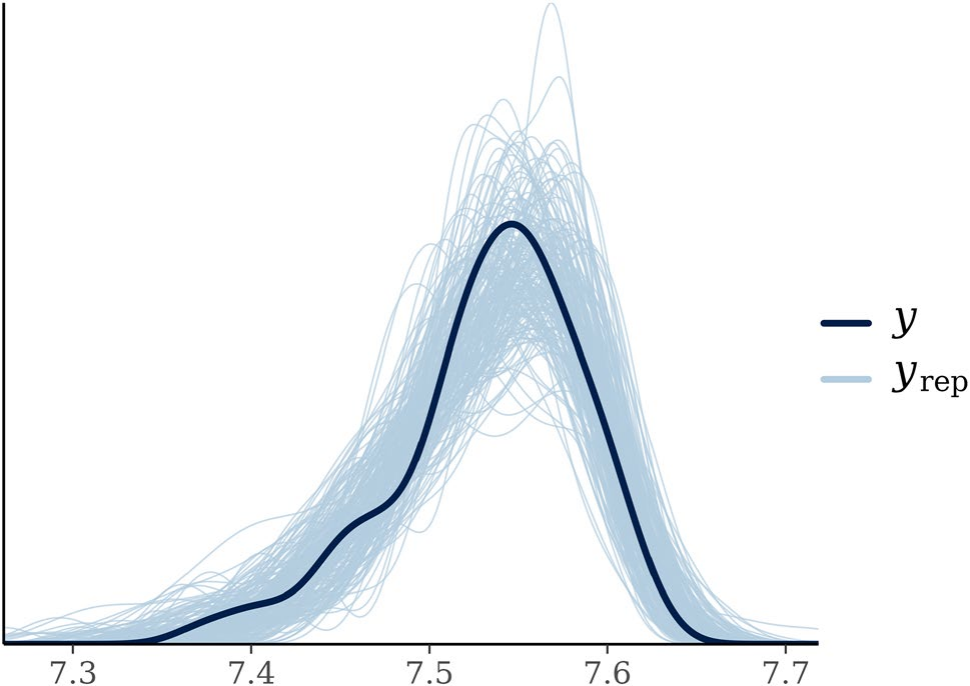
Kernel density estimation of the marginal probability density function of variable pH (dark thick black) is compared with 200 estimates of the same density function based on samples from the predictive posterior distribution (light grey).

Using the LOO criterion, the skew-normal distribution was selected for variable pH (pretreatment). In Fig. 2, 200 draws from the posterior predictive distribution have been performed and the correspondent estimates of the pdf are shown in light grey, where the thick black line is the estimate based on the collected sample.

Using 5000 draws from the posterior predictive distribution, the first and last percentile were calculated, together with realized min and max values. Table 2 below contains the inferred endpoints of intervals to recommend inspection of extreme points. The complete data can be found in Table 2S (Supplementary material).

**Table 2:** Body state descriptor variables with estimated threshold values; Q01_pr (estimated first percentile, left endpoint), Q99_pr (estimated last percentile, right endpoint).

Name	Q01_pr	Q99_pr
pH	7.38	7.63
pCO_2_	16.10	35.06
pO_2_	6.83	173.80
Na_p	57.15	95.94
K_p	1.64	3.89
Cl_m	58.47	89.55
Ca_pp	2.31	5.13
TCO_2_	13.80	28.50
nCa	2.62	5.37
pH_TC	7.38	7.88
pCO_2__TC	7.24	32.48
pO_2__TC	3.39	154.27
SBC	17.32	31.73
HCO_3__m	12.17	27.95
A	104.84	163.60
Osm	119.96	189.28

### Variable pH: the hypothesis of null treatment effect

**Figure 3:**
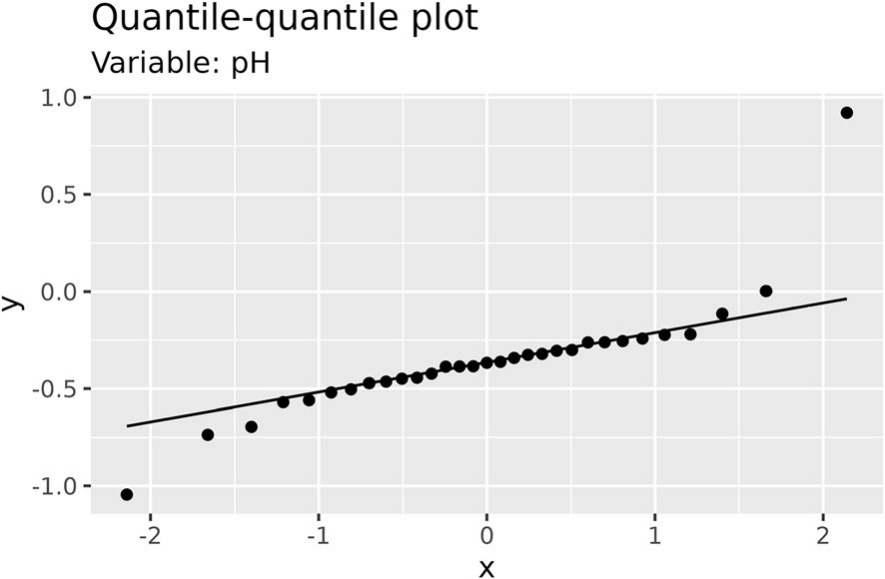
Quantile–quantile plot of variable pH.

In Fig. 3, the quantile–quantile plot of post–pre treatment differences are shown to appreciate possible departures from normality.

After removing an observation (top right), the above mentioned three models (Normal, Student t, Skew Normal) were fitted and the posterior distribution of ${\mu }_{D,1}$ (the mean value of the difference due to treatment) for the best model was estimated by MCMC simulation: the Student-t model was selected by the LOO criterion and in particular no sampled value was greater than zero, i.e. the estimated probability is $\widehat{P}\left[{\mu }_{D,1}>0\right]<0.002$. Tail probabilities for ${\mu }_{D,i}$ are shown in Table 3.

**Table 3:** Final models selected for each variable that describe body state.

Name	Model	Q01_diff	Q99_diff	less0
pH	Student t	− 0.46	− 0.29	< 0.0002
pCO_2_	Normal	76.33	133.07	> 0.9998
pO_2_	Skew normal	− 42.76	− 8.75	< 0.0002
Na_p	Skew normal	− 10.32	− 1.20	0.0036
K_p	Skew normal	− 0.06	0.91	0.9812
Cl_m	Skew Normal	− 12.92	− 5.03	2e-04
Ca_pp	Skew normal	0.88	3.76	0.9986
TCO_2_	Student t	19.62	36.27	> 0.9998
nCa	Skew normal	0.44	3.07	0.998
pH_TC	Student t	− 0.52	− 0.33	< 0.0002
pCO_2__TC	Normal	47.93	90.40	> 0.9998
pO_2__TC	Normal	− 34.70	4.45	0.0318
SBC	Student t	3.82	15.99	> 0.9998
HCO_3__m	Student t	16.53	31.48	> 0.9998
A	Normal	− 88.60	− 42.85	< 0.0002
Osm	Skew normal	− 19.07	− 2.52	0.0018

### Stun induction

In terms of stun induction, all snails immersed in CO_2_-enriched water were shown to achieve an acceptable level of unconsciousness, resulting in the stunning outcome described above.

## Discussion

In this pilot study, we proposed and investigated an original stunning method based on water supplemented with CO2 that could be used safely and humanely in the snail meat production chain. A Bayesian statistical analysis revealed substantial changes in many hemolymph components, like pH, Na, and Cl, in snails after immersion in gaseous water for a time sufficient to get them stunned. This finding is consistent with the literature-supported hypothesis that hypercapnia allows these animals to enter a dormant state. Furthermore, plausible physiological ranges, outside of which observed values should be investigated for the potential presence of large measurement errors and/or atypical responses of snails to treatment, were identified through analysis of the measured basal values of hemolymph parameters.

From a physiological perspective, the exposure of snails to cold and gaseous water facilitates the induction of torpor in snails, as well as a physiological response, as evidenced by changes in hemolymph components, confirming that CO_2_ has an effect on mollusks’ metabolism, depressing it and driving them to unconsciousness in a relatively short period of time. A remarkable feature of this procedure is its reversibility: even when applied several times to snails, they are able to recover without side effects. For that reason, too, this method of stunning can be considered humane for animals. Another major advantage is that it is possible to perform group stunning, which is a significant asset in favor of the future adoption of this method in industrial practice.

From the statistical viewpoint, this is an initial study that should be followed by extensive experimentation to verify if a better family of distributions could be used, for example one in which the sample space is bounded, i.e., it does not reach infinity.

As to the methodology to measure the components of hemolymph, hemogasanalysis appeared to be a useful and quick tool, as well as suitable, since hemolymph is an equivalent of mammalian blood tissue. Nevertheless, a future, comparative study of alternative measurement methods could be planned, to determine the best procedure for measuring hemolymph components.

In conclusion, it can be stated that the availability of a simple, low-cost, soft stunning method that can be applied on a small to large scale could benefit the welfare of millions of snails that enter the food chain each year. Indeed, these animals that are classifiable as sentient beings would be saved from death by boiling while still alive and conscious. The results of the present study confirm that immersion in CO_2_-enriched cold water can render them insensible in a short period of time without causing distress, as the snail lung can adapt to aquatic respiration and hypercapnia slows their metabolism, inducing a state of unconsciousness.

This study also identified basal values for several measurable parameters in snail hemolymph, which are useful for determining a physiological range for these parameters and can be refined with future research.

Although this is a preliminary research, the statistical analysis performed allowed for the collection of results that encourage the study to be continued and expanded in order to confirm its suitability and possibly promote the creation of specific guidelines for the humane slaughter of land snails, as even the AVMA Guidelines for the Humane Slaughter of Animals (2016 Edition)^27^ still do not include such species, although the same association lists some “acceptable with conditions methods” to anesthetize them before euthanasia, confirming the importance of stunning these sentient invertebrates (AVMA Guidelines for the Euthanasia of Animals, 2020 Edition)^28^.

## Animals and methods

**Figure 4:**
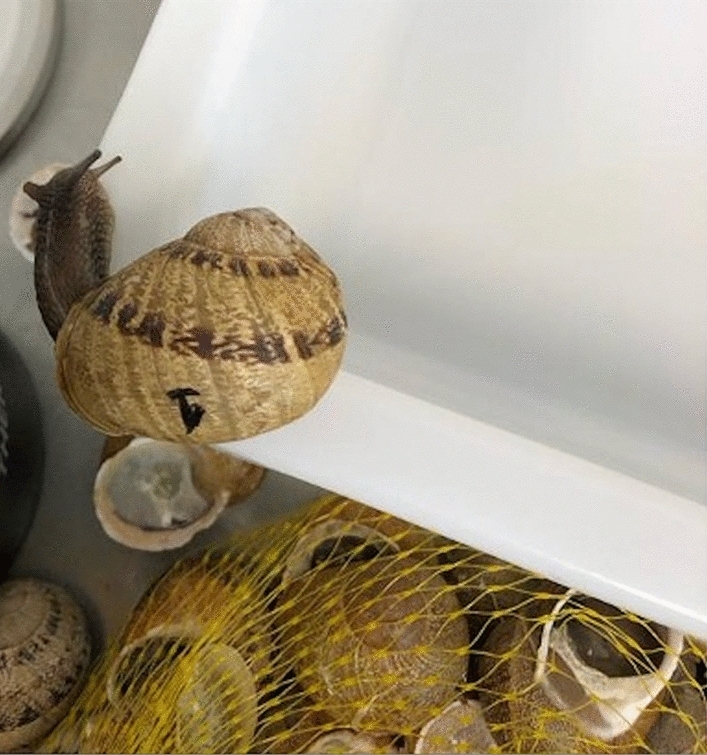
Snails placed in a clean, plastic container.

**Figure 5:**
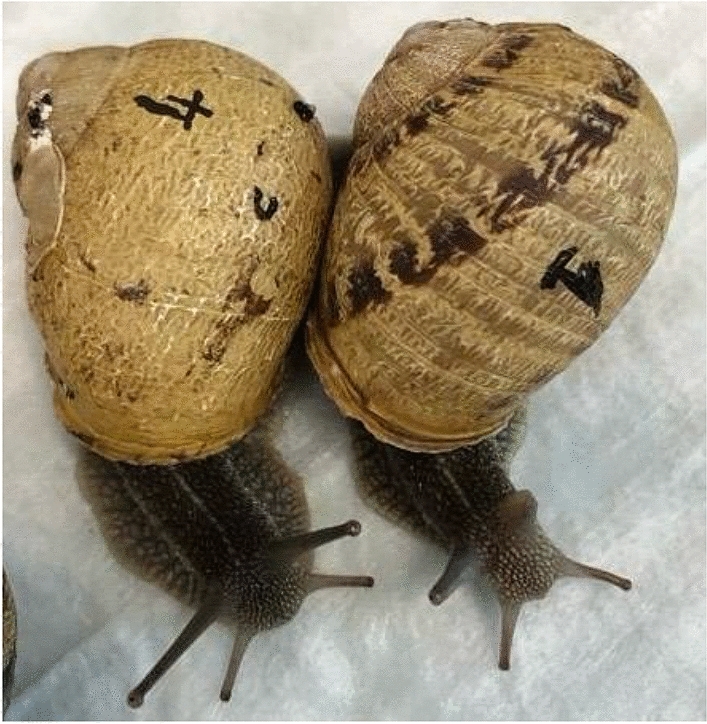
Snails with ID number.

**Figure 6:**
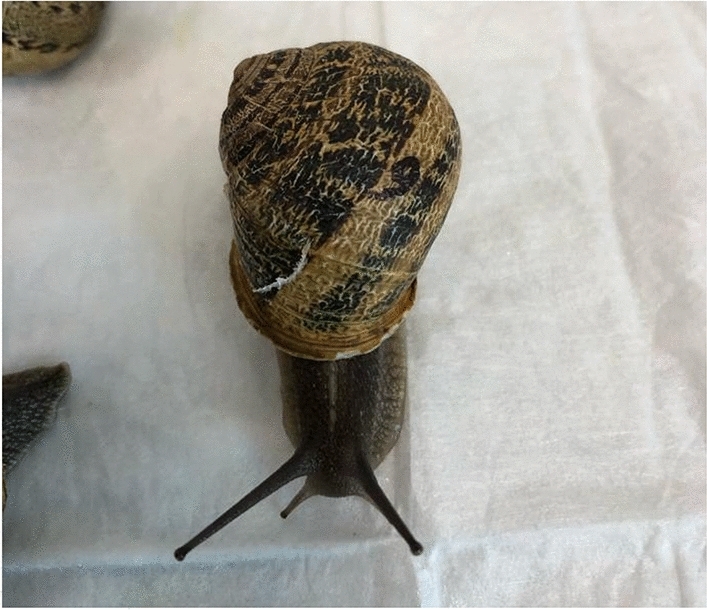
Snail with ID number.

The research was carried out on a sample of 75 *Cornu aspersum* (weight: 10–12 g) alive and vital, purchased from a local retail market where the vendor was directly a local snail farmer. The animals were placed in clean plastic containers (Fig. 4), exposed to a natural light–dark cycle, and kept under environmental temperature and humidity conditions similar to those present in the source area, as the farm of origin was located not far from the research facility. No nutrition was provided to maintain the same conditions in which the snails are kept ready to be prepared as food. Each snail was identified by drawing an indelible ID number on each shell (Figs. 5 and 6).

**Figure 7:**
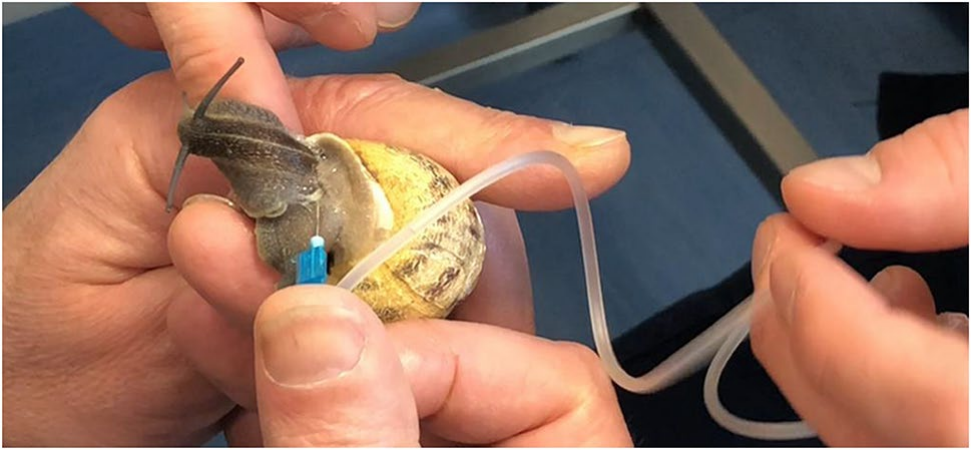
Sampling of hemolymph from snails.

**Figure 8:**
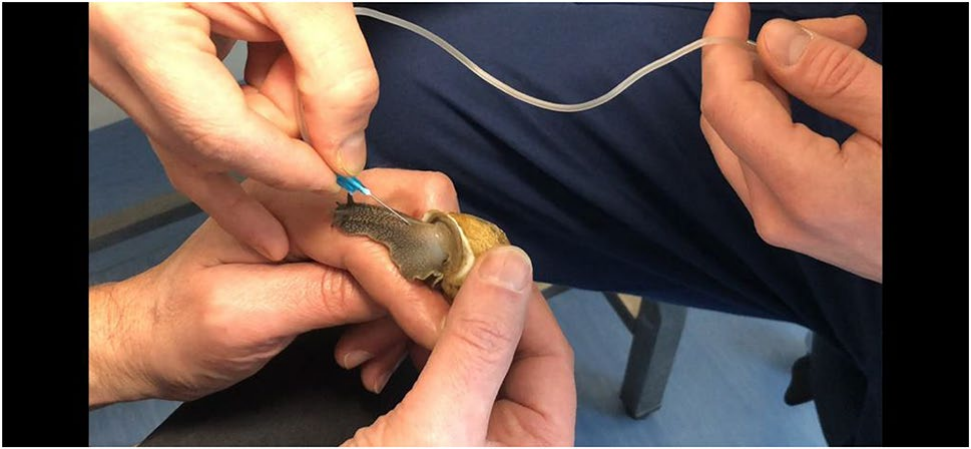
Sampling of hemolymph from snails.

**Figure 9:**
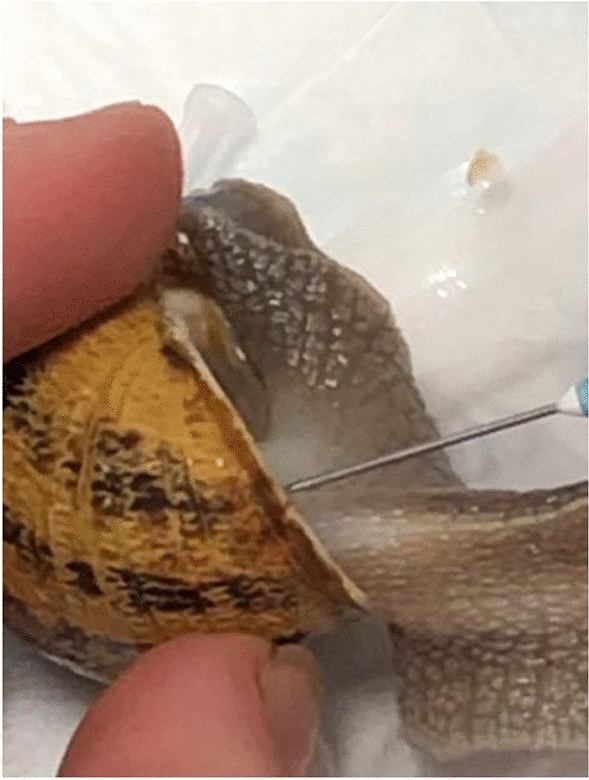
Sampling of hemolymph from snails.

**Figure 10:**
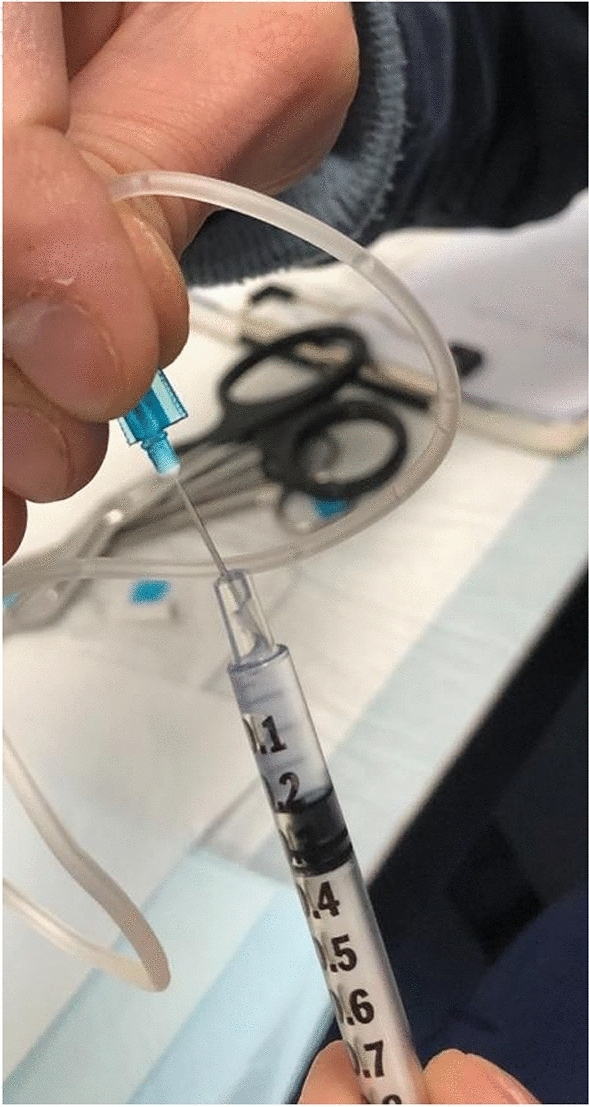
Transfer of hemolymph into a sterile insulin syringe.

In the first phase of the study, snails were subjected to hemolymph sampling (Figs. 7, 8 and 9), using the method described by J.E. Cooper^29^. Hemolymph sampling was carried out through the sinus region with a sterile butterfly needle (G23 X ¾) and 300mm length tubing. The method does not require the sacrifice of the mollusk and does not determine death or long-term negative impacts on the health of the animal, so as to allow the periodic extraction of hemolymph on the same source animal, if needed. The volume of hemolymph collected from each snail ranged between 0.3 and 0.6 ml. The freshly drawn hemolymph was immediately transferred into a sterile insulin syringe (Fig. 10) and analyzed, using the IDEXX VetStat® Electrolyte Blood Gas Analyzer for veterinary use, which measured the following sixteen parameters: acidity (pH), carbon dioxide partial pressure (pCO_2_), oxygen partial pressure (pO_2_), sodium concentration (Na^+^), potassium concentration (K^+^), chlorine concentration (Cl^−^), ionized calcium concentration (Ca^++^), carbon dioxide concentration (TCO_2_), (ionized calcium normalized to PH 7.4) nCa, transcutaneous acidity (pH(TC)), transcutaneous carbon dioxide partial pressure (pCO_2_ (TC)), transcutaneous oxygen partial pressure (pO_2_ (TC)), standard bicarbonate concentration (SBC), bicarbonate ion concentration (HCO_3_^−^), Alkalosis (A), osmolarity (Osm). These values were statistically processed in order to determine the plausible physiological range of each variable.

In the second phase of the study, a quota of the specimens was tested for stunning by immersion in mineral drinking water enriched with a high concentration (10 g/l) of food-grade CO_2_ (E290) and maintained at 6–8 °C. This step of the procedure was aimed at assessing their sensitivity to CO_2_-supplemented and refrigerated water and to observe their reaction to the point of verifying the induction of their stunning. The temperature of the water was chosen to match the temperature at which the snails are typically kept in order to preserve them before industrial slaughter. The low temperature also helps to maintain the level of dissolved CO_2_ in the water, as CO_2_ solubility is temperature dependent and is better maintained in cold water. Furthermore, it has been found that temperatures below 10°C induce and maintain torpor in snails, as is the case during their winter dormancy^30^.

**Figure 11:**
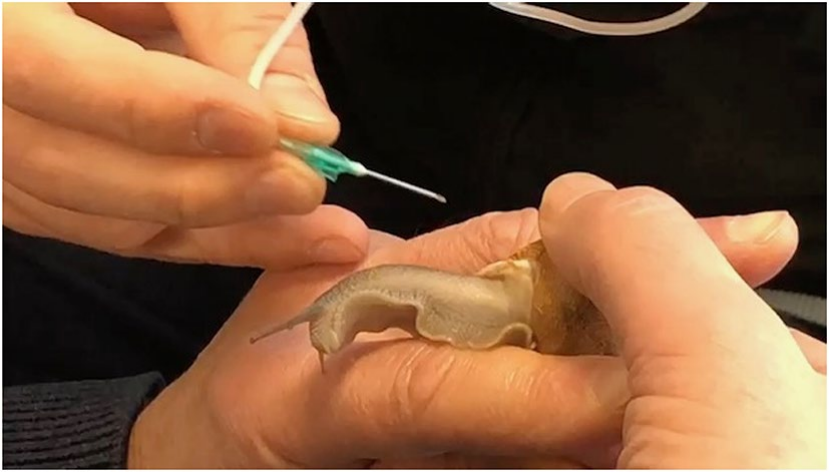
Sampling of hemolymph from a stunned snail.

The surface of the water was covered with a plastic contact film to limit the dispersion of CO_2_ into the air during the test. The snails were immersed completely in water and observed while the immersion time was recorded. The expected outcome of stunning was identified as follows: an animal that was completely out of its shell and relaxed, with no reactivity to even the stimulus of the eye tentacles. The time required to stun each snail was recorded by a trained researcher. It ranged from 4 to 5 min. After the stunned condition was met, hemolymph sampling was repeated (Fig. 11), and gas measurements were taken immediately. The snails used to test the effect of carbonated water immersion were chosen at random from those used for the first hemolymph sampling. The ID number drawn on each shell made it possible to compare the outcomes of the same snail before and after treatment with CO_2_-supplemented water.

No deaths were observed in any of the groups of animals (before and after stunning) during the study.

When kept out of water, after they were stunned, all the snails recovered a vital condition in 9 to 15 min.

### Statistical analysis

Sixteen variables describing body state of snails have been considered among those made available by a common measurement equipment. Summary statistics conditional on treatment level were calculated before and after removing outliers for each variable: number of observations, minimum, mean, standard deviation, median, maximum and the first and third quartiles. Outliers were identified by calculating an interval $\left({l}_{1},{l}_{2}\right)$ outside which the values were declared extreme outliers^31^, that is

$${l}_{1}={Q}_{1}-3\cdot \left({Q}_{3}-{Q}_{1}\right)$$

$${l}_{2}={Q}_{3}+3\cdot \left({Q}_{3}-Q1\right)$$

With ${Q}_{1}$ the first and ${Q}_{3}$ the third quartiles.

Three Bayesian models were fitted to each variable ${Y}_{i},i=\mathrm{1,2},\dots ,16$ measured before treatment using samples $\left({y}_{i,1},\dots ,{y}_{i,j},\dots ,{y}_{i,{n}_{i}}\right)$. The considered families of probability density functions were:

the Normal

$$p\left(y |\boldsymbol{ }\mu ,{\sigma }^{2}\right)={\text{Normal}}\left(\mu ,{\sigma }^{2}\right)=\frac{1}{\sqrt{2\pi {\sigma }^{2}}}exp\left(\frac{-1}{2}\frac{{\left(y-\mu \right)}^{2}}{{\sigma }^{2}}\right)$$

the Student-t

$$p\left(y | \mu ,\sigma ,\nu \right)=\text{Student-t}\left(\mu ,\sigma ,\nu \right)=\frac{\Gamma \left(\left(\nu +1\right)/2\right)}{\Gamma \left(\nu /2\right)\sqrt{\nu \pi \sigma }}{\left(1+\frac{1}{\nu }{\left(\frac{y-\mu }{\sigma }\right)}^{2}\right)}^{-\left(\nu +1\right)/2}$$

and the Skew Normal family

$$p\left(y | \xi ,\sigma ,\omega ,\alpha \right)=\text{Skew-Normal}\left(\xi ,\sigma ,\omega ,\alpha \right)=\frac{1}{\sqrt{2\pi \sigma }}exp\left(\frac{-1}{2}{\left(\frac{y-\xi }{\omega }\right)}^{2}\right)\left(1+erf\left(\alpha \left(\frac{y-\xi }{\omega \sqrt{2}}\right)\right)\right)$$

Model selection was performed after model comparison based on the LOO criterion (Leave-One-Out^32^). A sample from the posterior distribution.

$p\left({\theta }_{i}| {y}_{i,1},\dots ,{y}_{i,{n}_{i}}\right)$ of model parameters was obtained for each pre-treatment variable by Markov Chain Monte Carlo simulation using *STAN* software^33,34^ in *R*^35^ and the *brms* R package^36^. A sample from the predictive distribution of each variable, say $p\left({y}_{f,i}| {y}_{i,1},\dots ,{y}_{i,{n}_{i}}\right)$ was also obtained by Monte Carlo simulation, then 1% and 99% percentiles were calculated to define reasonable threshold values outside which future measurements should be checked for being atypical or potentially affected by strong measurement errors.

A similar modeling exercise was performed for the difference after—before treatment. Let ${D}_{i,j}={Y}_{a,i,j}-{Y}_{b,i,j}$ be the difference of variables referring to body feature $i$ after ($a$) vs before ($b$) treatment with CO_2_, with $i=,1,\dots ,16$ the index denoting variables and $j=\mathrm{1,2},\dots ,{n}_{i}$ the index for observations. The statistical null hypothesis that treatment has no effect follows from

$${D}_{i,j}\sim N\left({\mu }_{D,i}=0,{\sigma }_{D,i}^{2}\right)=\frac{1}{\sqrt{2\pi {\sigma }_{D,i}}}exp\left(\frac{-1}{2}{\left(\frac{{d}_{i,j}-{\mu }_{D,i}}{{\sigma }_{D,i}}\right)}^{2}\right)$$

thus ${H}_{0}:{\mu }_{D,i}=0$ vs ${H}_{1}:{\mu }_{D,i}\ne 0$ for the expected value of variable ${D}_{i}$. The null hypothesis was rejected when the marginal posterior distribution of ${\mu }_{D,i}$ was located far away from zero, that is $P\left[{\mu }_{D,i}>0\vee {d}_{i,1},\dots ,{d}_{i,{n}_{i}}\right]$ was close to zero or close to one, say greater than $0.99$ or smaller than $0.01$.

### Ethics declarations

Although the study involved invertebrates and was not harmful to the animals, it received ethical oversight by the Animal Welfare Body (OPBA) of the Università degli Studi di Milano, which approved it.

### Supplementary Information

Supplementary Tables.

Supplementary Figures.

## Data Availability

The datasets used and/or analyzed during the current study are reported in the article.
