# Supplementary material for: Welfare of invertebrates: a pilot study on a new land snail stunning technique
Source: Sci Rep. 2024 Apr 10;14:8378. doi: 10.1038/s41598-024-58133-4 (PMC11006846; doi:10.1038/s41598-024-58133-4)
Supplement: Supplementary file 1 — Supplementary Tables. [file 41598_2024_58133_MOESM1_ESM.docx]

**aSupplementary materials**

**Table 1S:** summary statistics of raw data for each variable given the selected treatment without removing extreme observations (outliers).

| Variable | Treatment | Min | Q.25 | mean | Q.50 | sd | Q.75 | Max | n_obs |
| --- | --- | --- | --- | --- | --- | --- | --- | --- | --- |
| pH | Bas | 6.46 | 7.51 | 7.52 | 7.54 | 0.13 | 7.57 | 6.46 | 74 |
| pH | CO2 | 6.54 | 7.06 | 7.15 | 7.21 | 0.21 | 7.26 | 6.54 | 31 |
| pCO2 | Bas | 15.60 | 21.60 | 25.30 | 23.50 | 7.96 | 26.25 | 15.60 | 74 |
| pCO2 | CO2 | 46.20 | 86.60 | 138.47 | 124.70 | 73.94 | 160.10 | 46.20 | 31 |
| pO2 | Bas | 32.50 | 63.70 | 92.00 | 93.60 | 35.26 | 119.10 | 32.50 | 74 |
| pO2 | CO2 | 20.70 | 40.10 | 63.97 | 50.00 | 35.07 | 78.15 | 20.70 | 31 |
| Na_p | Bas | 60.10 | 72.35 | 78.01 | 76.10 | 9.57 | 81.50 | 60.10 | 74 |
| Na_p | CO2 | 58.10 | 67.60 | 71.85 | 72.20 | 6.15 | 75.50 | 58.10 | 31 |
| K_p | Bas | 1.83 | 2.02 | 2.36 | 2.33 | 0.50 | 2.54 | 1.83 | 74 |
| K_p | CO2 | 1.98 | 2.19 | 2.83 | 2.34 | 1.22 | 2.76 | 1.98 | 31 |
| Cl_m | Bas | 61.30 | 70.55 | 74.81 | 74.40 | 7.33 | 76.95 | 61.30 | 74 |
| Cl_m | CO2 | 56.90 | 63.00 | 67.27 | 67.20 | 5.91 | 68.50 | 56.90 | 31 |
| Ca_pp | Bas | 2.73 | 3.41 | 3.73 | 3.74 | 0.54 | 4.12 | 2.73 | 74 |
| Ca_pp | CO2 | 3.47 | 4.90 | 6.29 | 6.28 | 1.98 | 7.22 | 3.47 | 31 |
| TCO2 | Bas | 13.40 | 19.60 | 21.59 | 21.40 | 4.15 | 22.70 | 13.40 | 74 |
| TCO2 | CO2 | 13.80 | 37.80 | 49.05 | 49.60 | 17.28 | 61.15 | 13.80 | 31 |
| nCa | Bas | 2.97 | 3.71 | 4.01 | 3.98 | 0.55 | 4.37 | 2.97 | 74 |
| nCa | CO2 | 3.64 | 4.15 | 5.38 | 5.25 | 1.44 | 6.26 | 3.64 | 31 |
| pH_TC | Bas | 6.46 | 7.62 | 7.70 | 7.75 | 0.18 | 7.81 | 6.46 | 74 |
| pH_TC | CO2 | 6.69 | 7.22 | 7.28 | 7.28 | 0.20 | 7.41 | 6.69 | 31 |
| pCO2_TC | Bas | 7.40 | 11.07 | 16.19 | 13.05 | 8.69 | 19.70 | 7.40 | 74 |
| pCO2_TC | CO2 | 22.00 | 56.40 | 87.05 | 75.30 | 42.61 | 113.90 | 22.00 | 31 |
| pO2_TC | Bas | 9.80 | 23.10 | 54.38 | 44.70 | 37.56 | 79.40 | 9.80 | 74 |
| pO2_TC | CO2 | 6.20 | 13.72 | 41.85 | 26.25 | 38.78 | 63.40 | 6.20 | 31 |
| SBC | Bas | 3.10 | 22.60 | 24.25 | 24.50 | 3.94 | 26.25 | 3.10 | 74 |
| SBC | CO2 | 5.10 | 27.75 | 35.43 | 33.60 | 14.47 | 40.65 | 5.10 | 31 |
| HCO3_m | Bas | 4.90 | 18.80 | 20.59 | 20.40 | 4.42 | 21.90 | 4.90 | 74 |
| HCO3_m | CO2 | 10.20 | 32.95 | 44.55 | 45.60 | 16.16 | 54.70 | 10.20 | 31 |
| A | Bas | 64.60 | 123.40 | 132.60 | 137.10 | 13.89 | 139.90 | 64.60 | 74 |
| A | CO2 | 5.70 | 40.20 | 67.58 | 63.50 | 37.39 | 92.95 | 5.70 | 31 |
| Osm | Bas | 125.10 | 145.50 | 156.04 | 152.50 | 17.68 | 162.00 | 125.10 | 74 |
| Osm | CO2 | 119.90 | 138.20 | 145.42 | 146.25 | 11.94 | 153.07 | 119.90 | 31 |

**Table 2S**: Body state descriptor variables with observed and estimated treshold values. Columns are: min_pr (predicted minimum by MCMC simulation), min_obs (observed minimum in the filtered sample), Q01_pr (estimated first percentile, left endpoint), max_obs (observed maximum value in the filtered sample), Q99_pr (estimated last percentile, right endpoint), max_pr (predicted maximum, by MCMC simulation).

| Name | min_pr | min_obs | Q01_pr | max_obs | Q99_pr | max_pr |
| --- | --- | --- | --- | --- | --- | --- |
| pH | 7.29 | 7.37 | 7.38 | 7.62 | 7.63 | 7.67 |
| pCO2 | 12.90 | 15.60 | 16.10 | 38.30 | 35.06 | 47.19 |
| pO2 | -47.46 | 32.50 | 6.83 | 163.20 | 173.80 | 234.03 |
| Na_p | 47.03 | 60.10 | 57.15 | 94.20 | 95.94 | 111.10 |
| K_p | 1.25 | 1.83 | 1.64 | 4.10 | 3.89 | 4.80 |
| Cl_m | 32.83 | 61.30 | 58.47 | 88.40 | 89.55 | 111.07 |
| Ca_pp | 1.88 | 2.73 | 2.31 | 4.72 | 5.13 | 6.87 |
| TCO2 | 10.08 | 13.40 | 13.80 | 27.80 | 28.50 | 33.33 |
| nCa | 1.76 | 2.97 | 2.62 | 4.95 | 5.37 | 6.15 |
| pH_TC | 7.19 | 7.37 | 7.38 | 7.87 | 7.88 | 7.96 |
| pCO2_TC | 4.38 | 7.40 | 7.24 | 37.20 | 32.48 | 45.61 |
| pO2_TC | -7.38 | 9.80 | 3.39 | 159.70 | 154.27 | 215.28 |
| SBC | 7.77 | 18.40 | 17.32 | 35.50 | 31.73 | 46.66 |
| HCO3_m | 0.42 | 13.00 | 12.17 | 30.80 | 27.95 | 44.32 |
| A | -5,659.10 | 101.90 | 104.84 | 179.30 | 163.60 | 282.35 |
| Osm | 12.12 | 125.10 | 119.96 | 186.80 | 189.28 | 239.80 |
