# Supplementary material for: Welfare of invertebrates: a pilot study on a new land snail stunning technique
Source: Sci Rep. 2024 Apr 10;14:8378. doi: 10.1038/s41598-024-58133-4 (PMC11006846; doi:10.1038/s41598-024-58133-4)
Supplement: Supplementary file 2 — Supplementary Figures. [file 41598_2024_58133_MOESM2_ESM.docx]

**Supplementary file, Fossati et al. 2024**


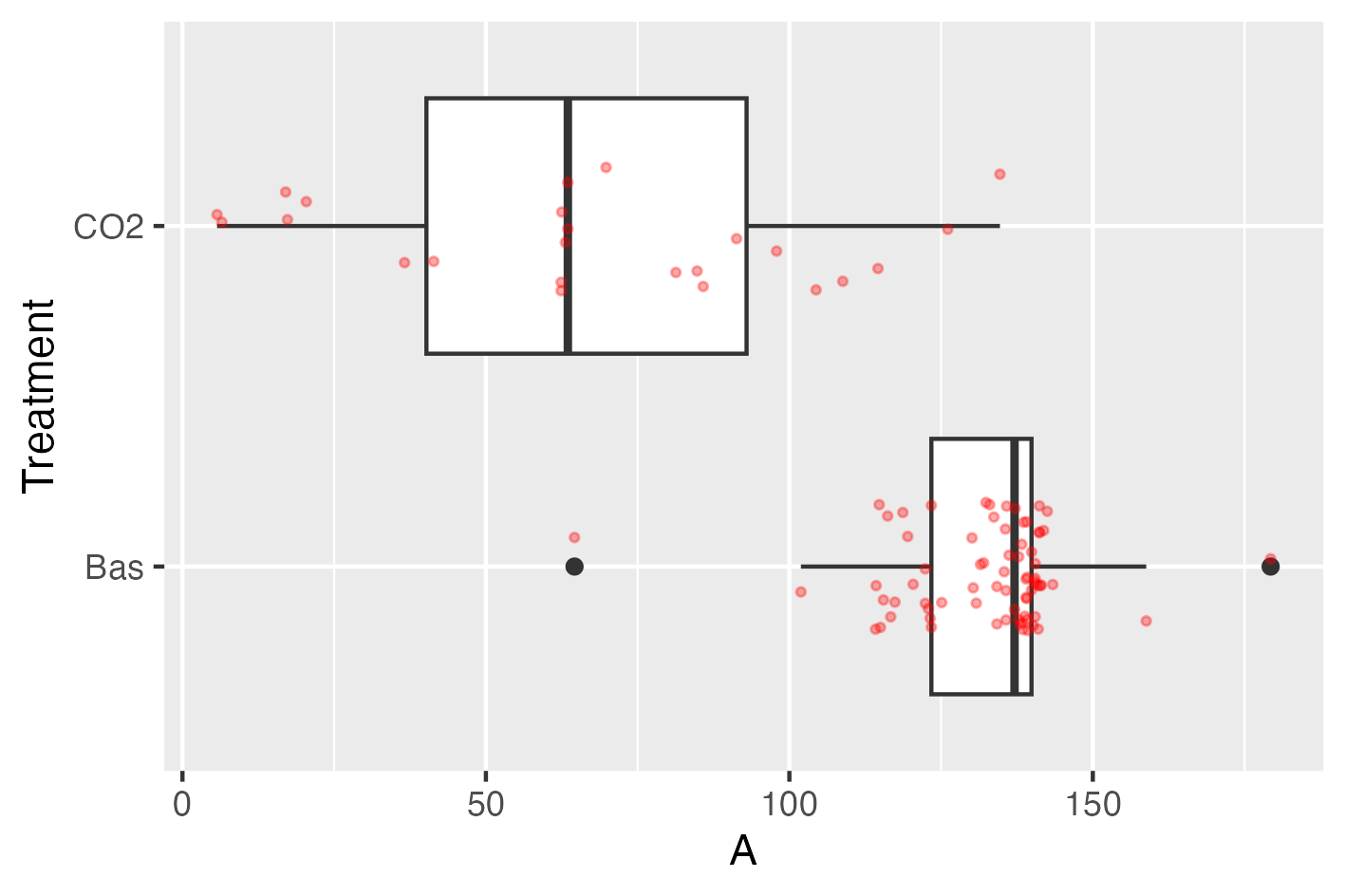


Fig. 1: Boxplots of variable A given treatment


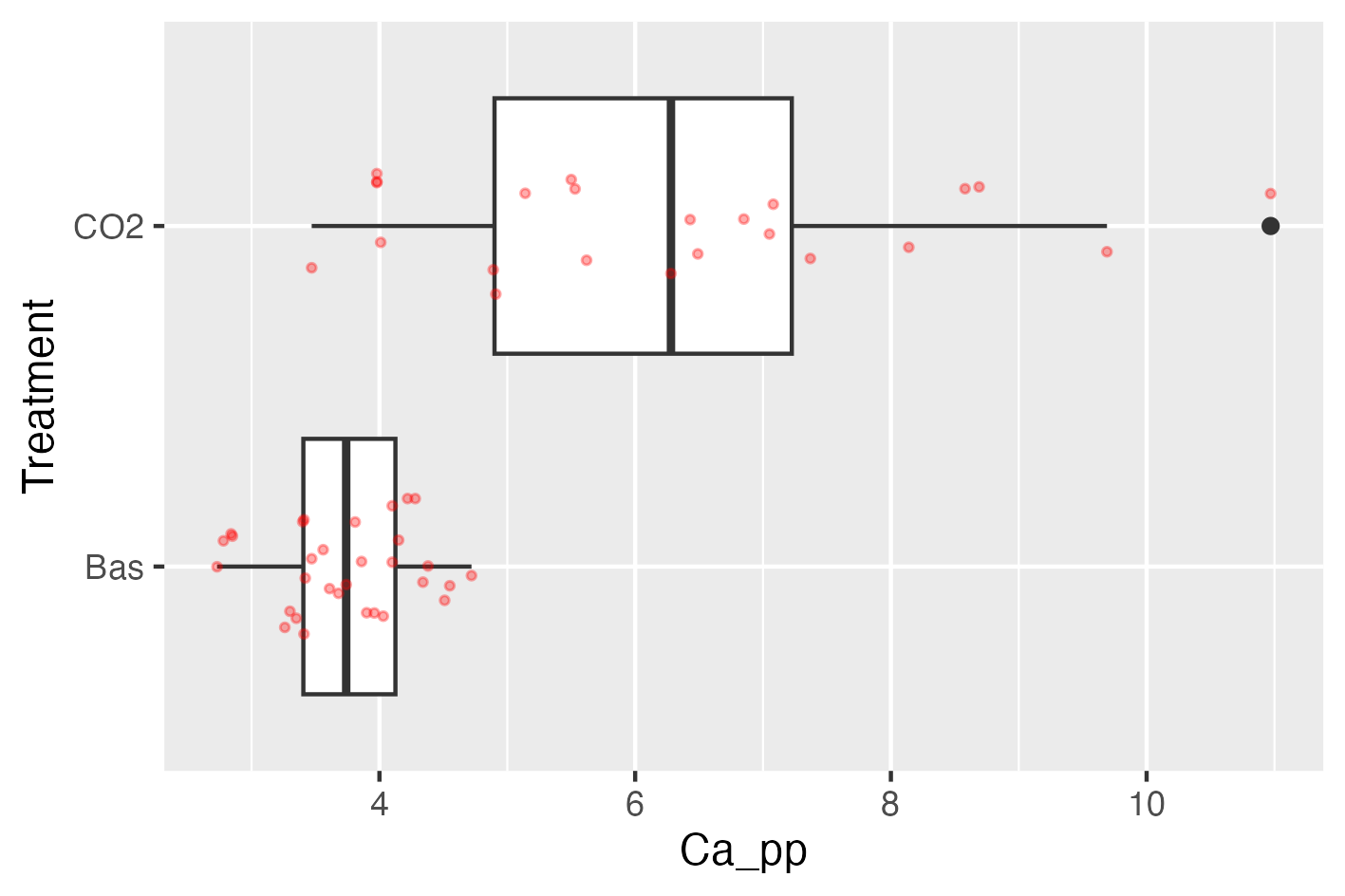


Fig. 2: Boxplots of variable Ca given treatment.


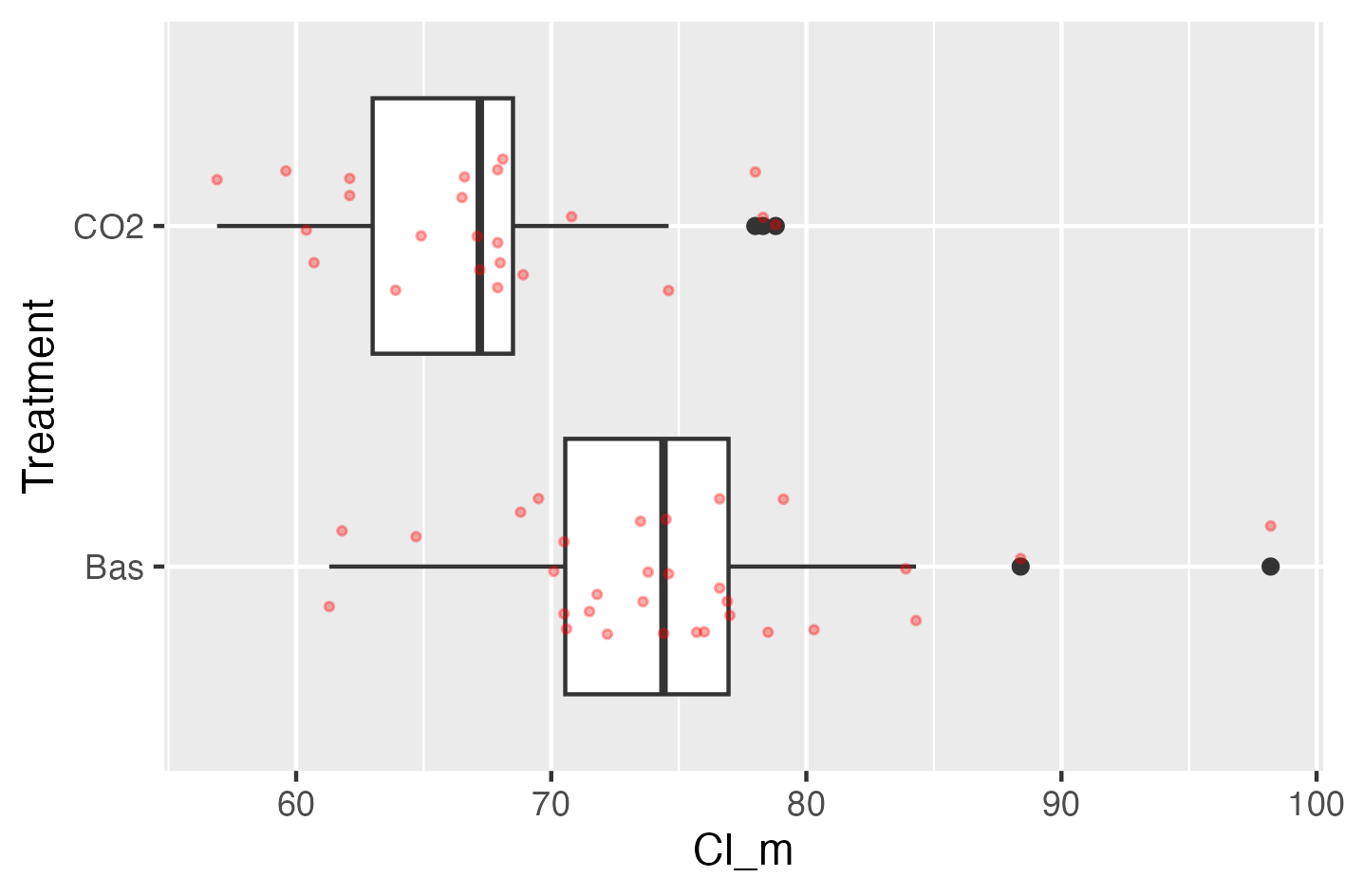


Fig. 3: Boxplots of variable Cl given treatment.


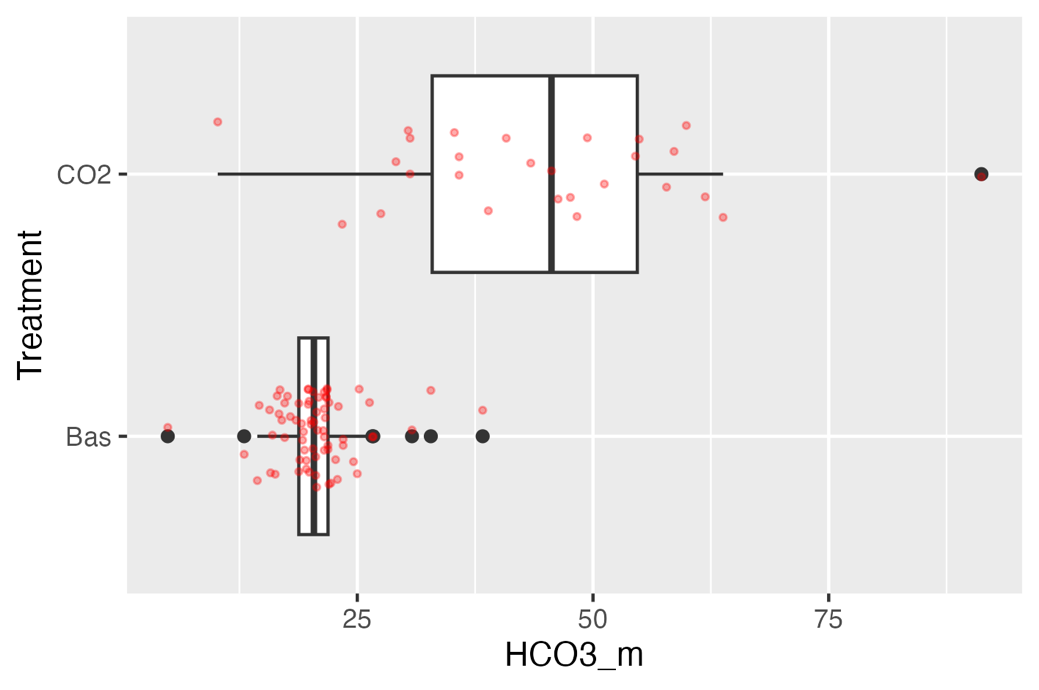


Fig. 4: Boxplots of variable HCO3 given treatment.


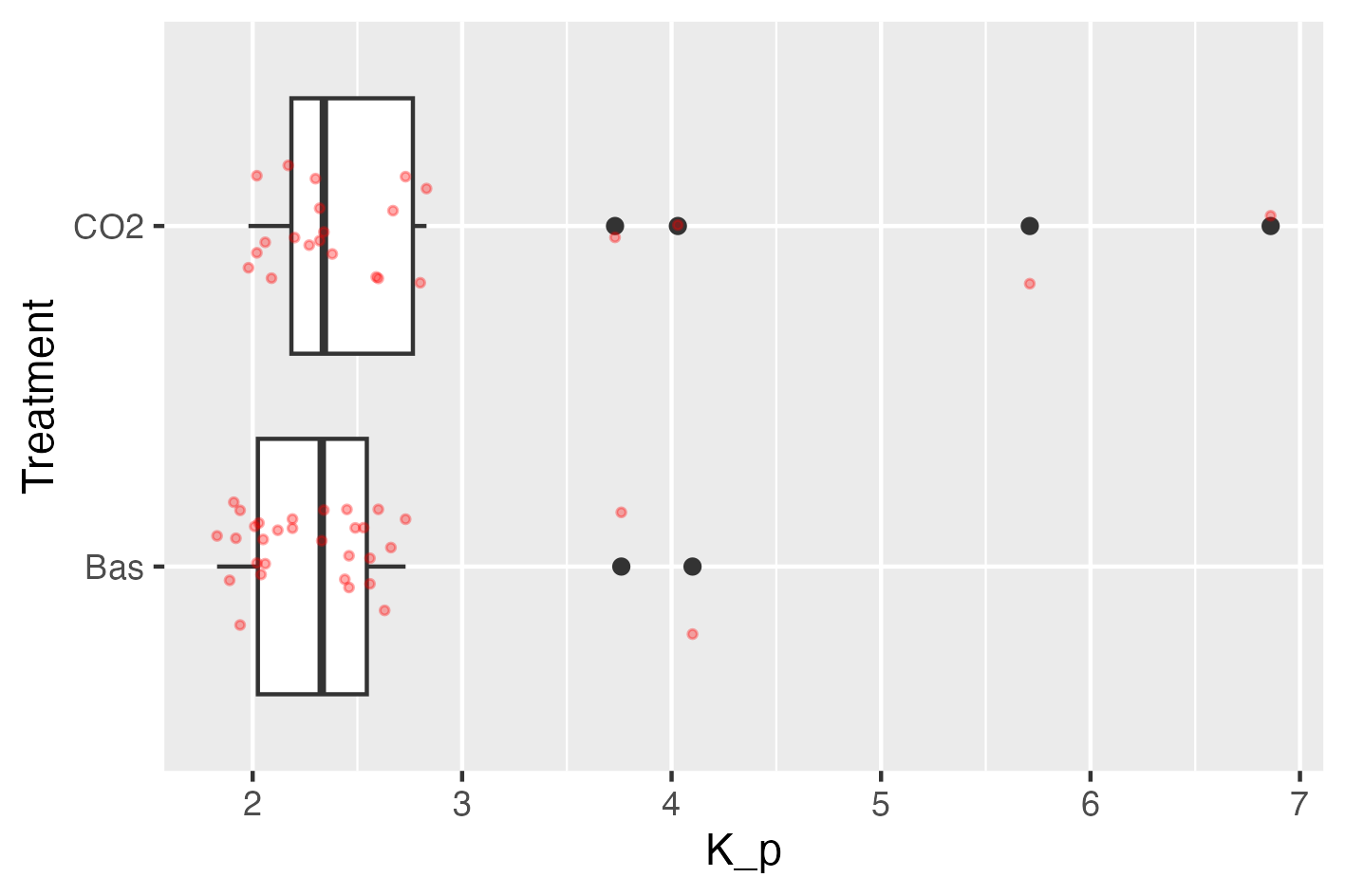


Fig. 5: Boxplots of variable K given treatment.


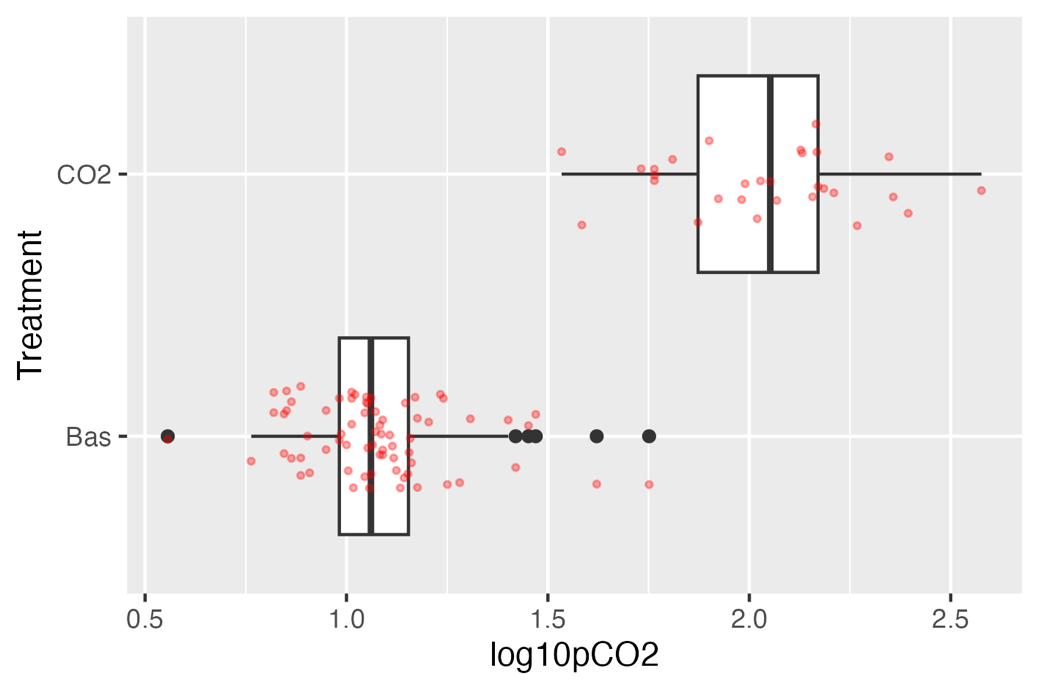


Fig. 6: Boxplots of variable pCO2 (natural log scale) given treatment.


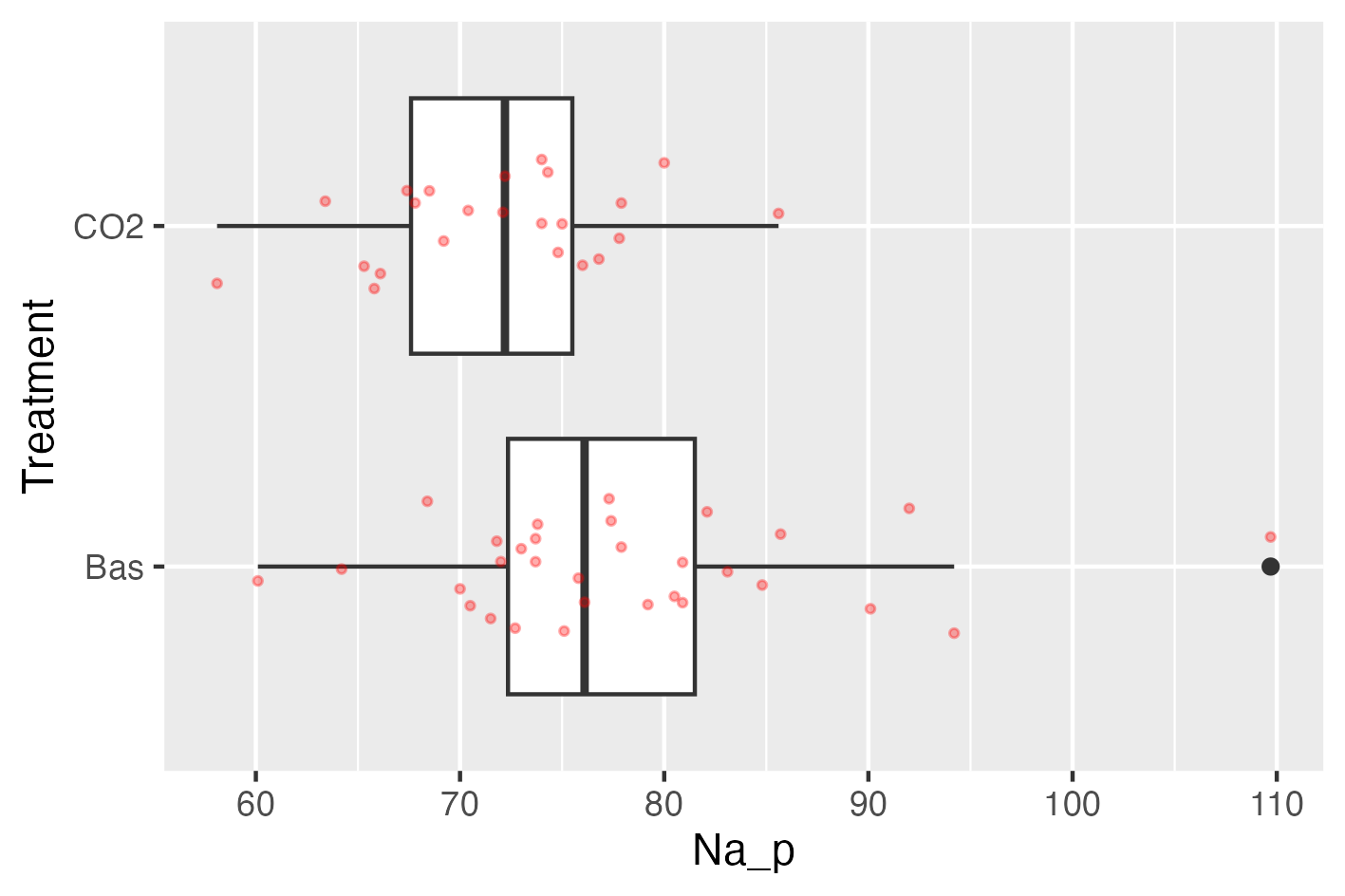


Fig. 7: Boxplots of variable Na given treatment.


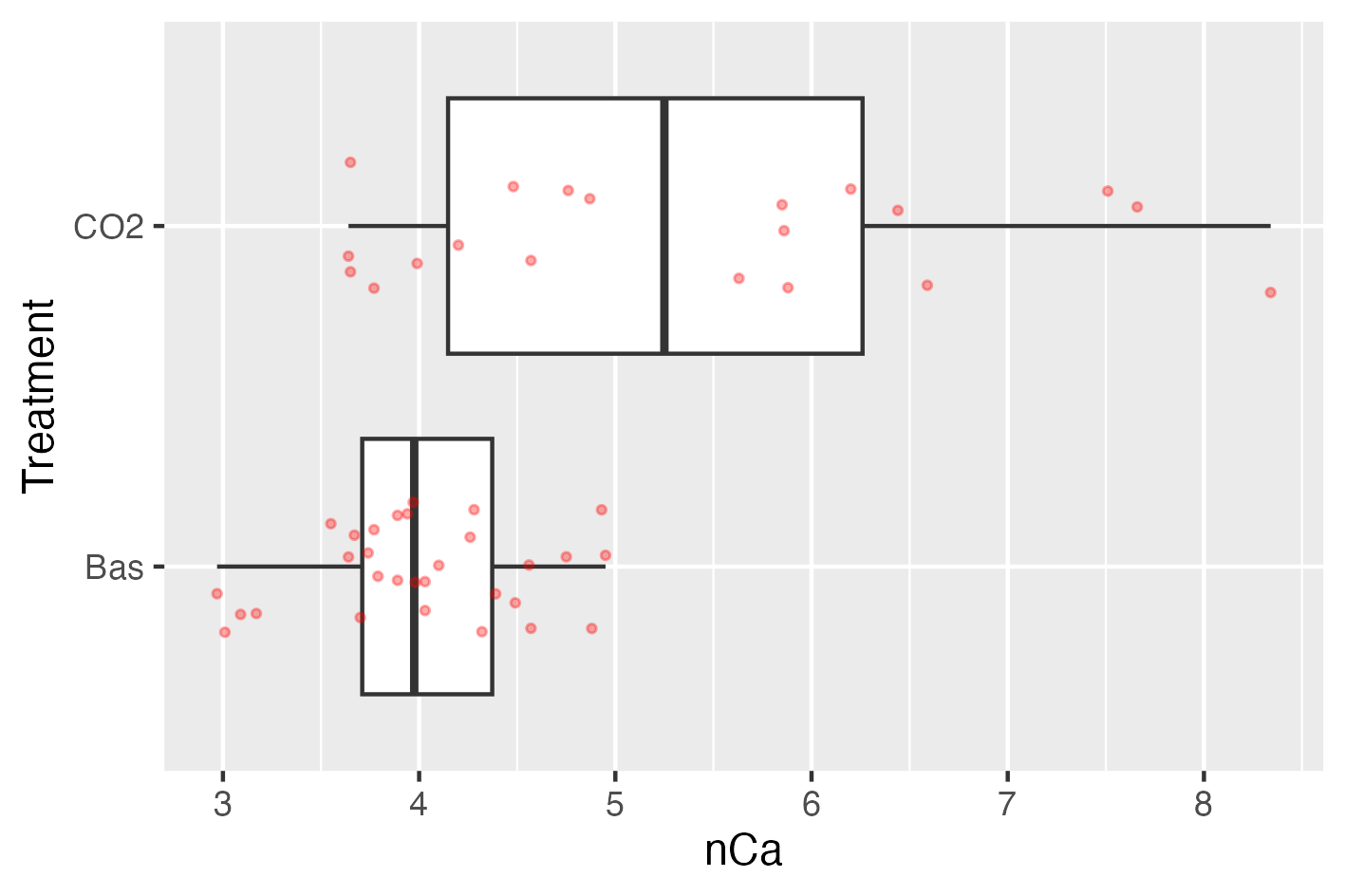


Fig. 8: Boxplots of variable nCa given treatment.


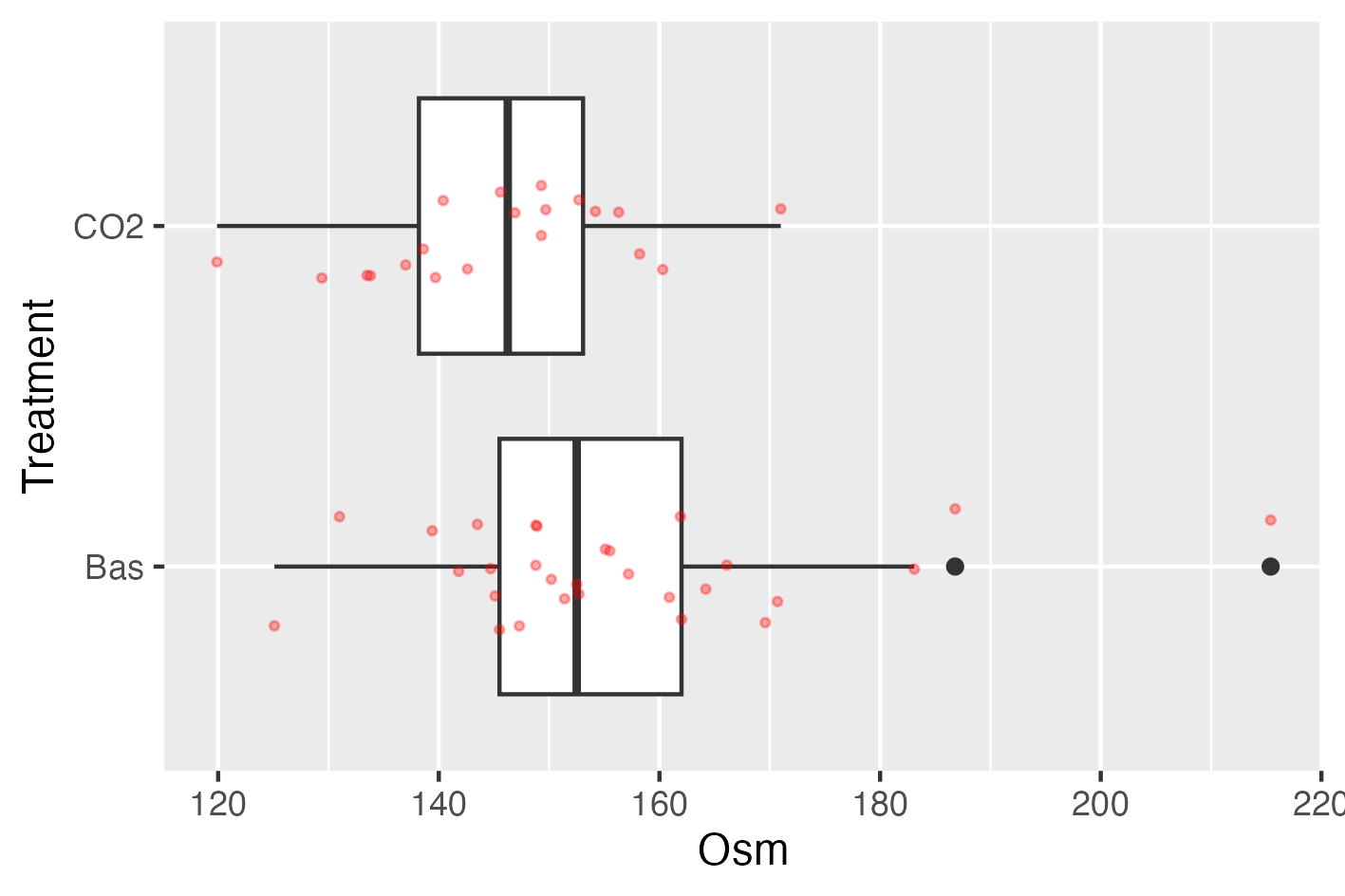


Fig. 9: Boxplots of variable Osm given treatment.


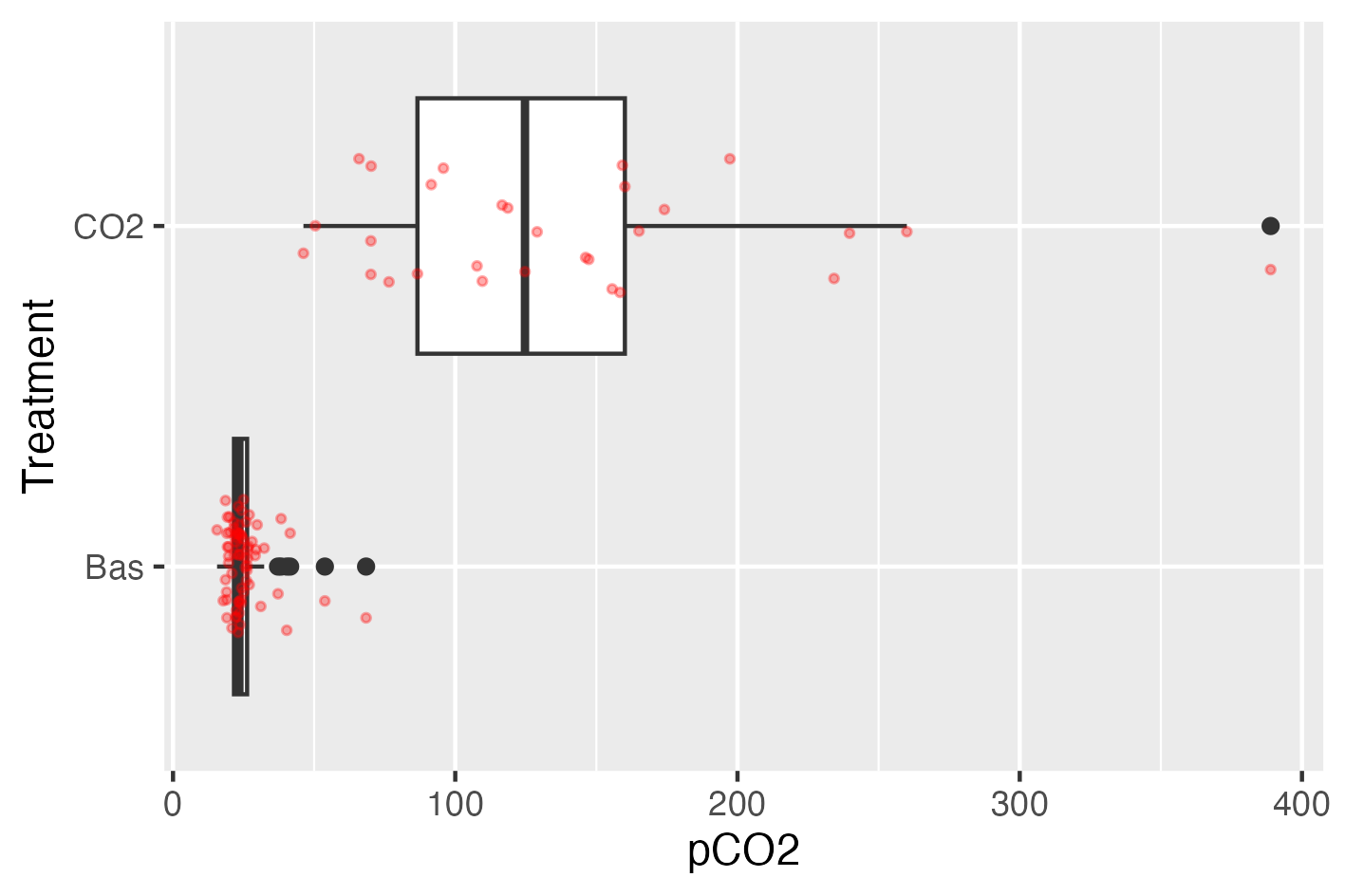


Fig. 10: Boxplots of variable pCO2 given treatment.


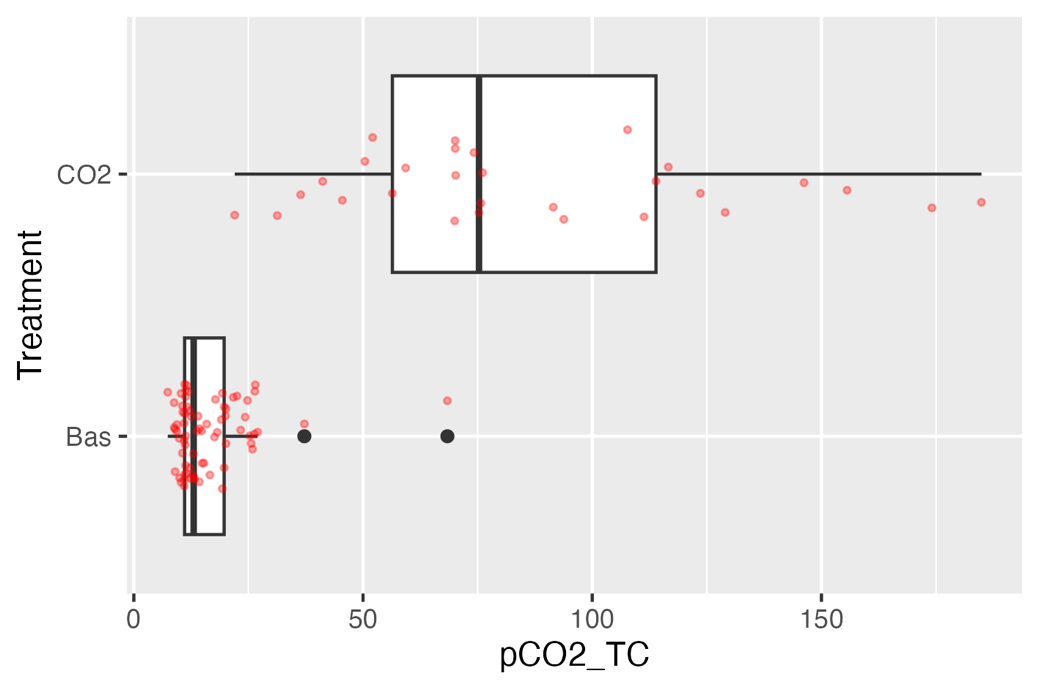


Fig. 11: Boxplots of variable pCO2_T given treatment.


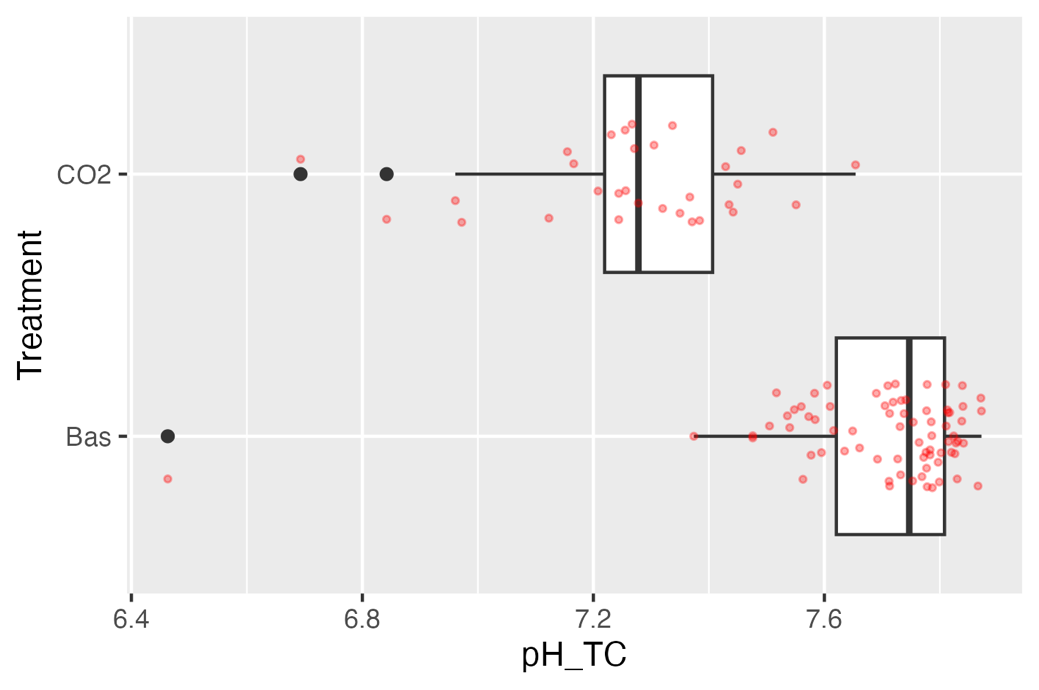


Fig. 12: Boxplots of variable pH_TC given treatment.


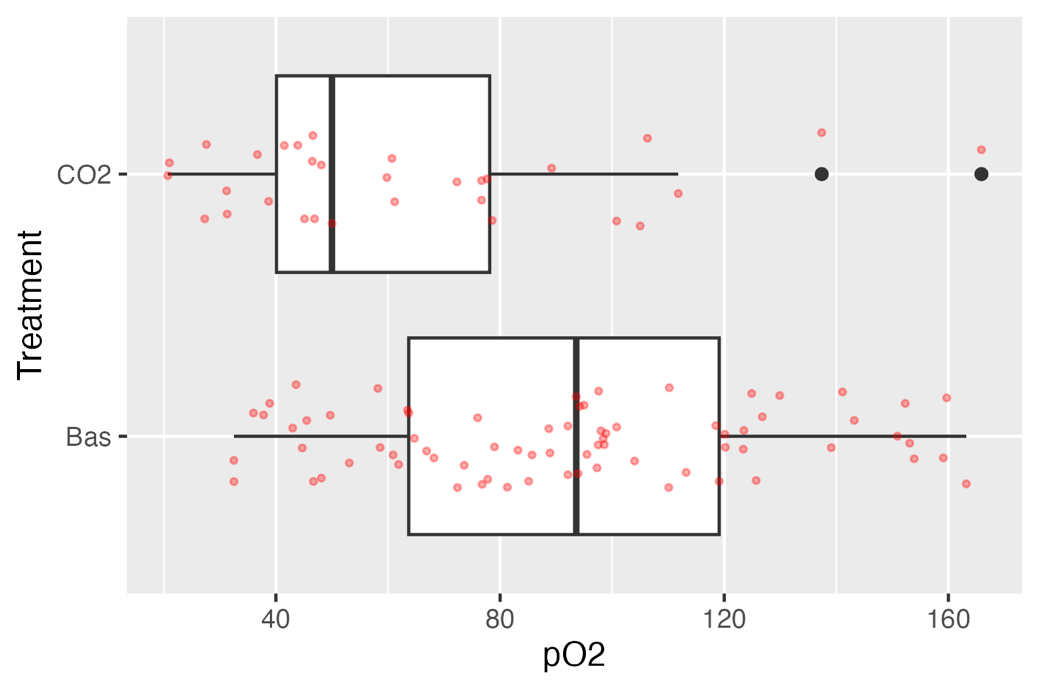


Fig. 13: Boxplots of variable pO2 given treatment.


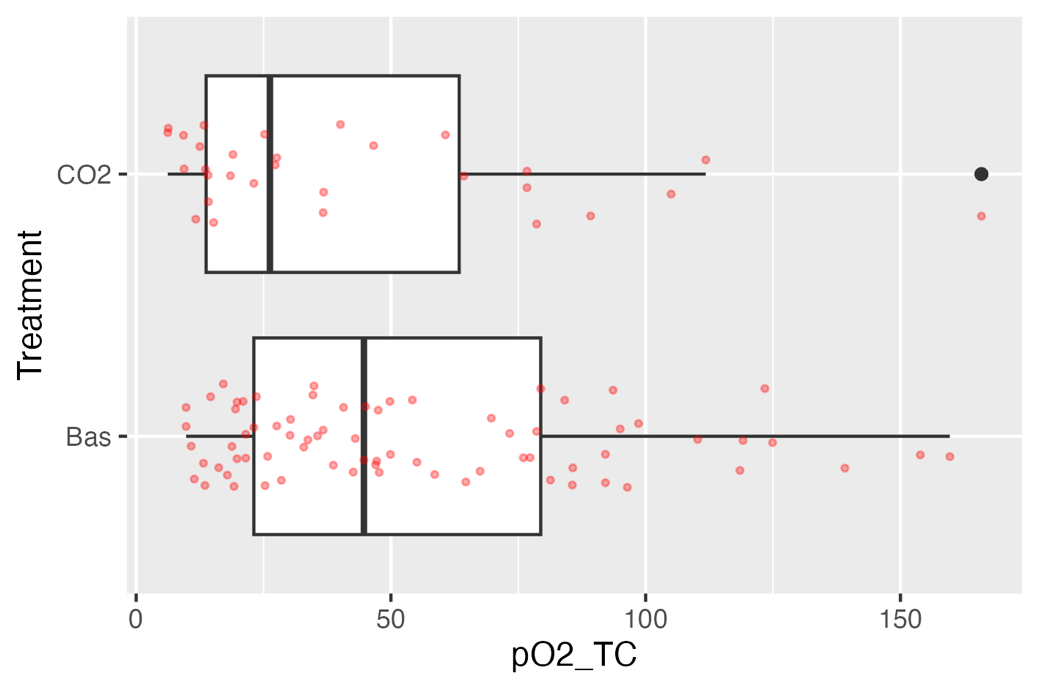


Fig. 14: Boxplots of variable pO2_TC given treatment.


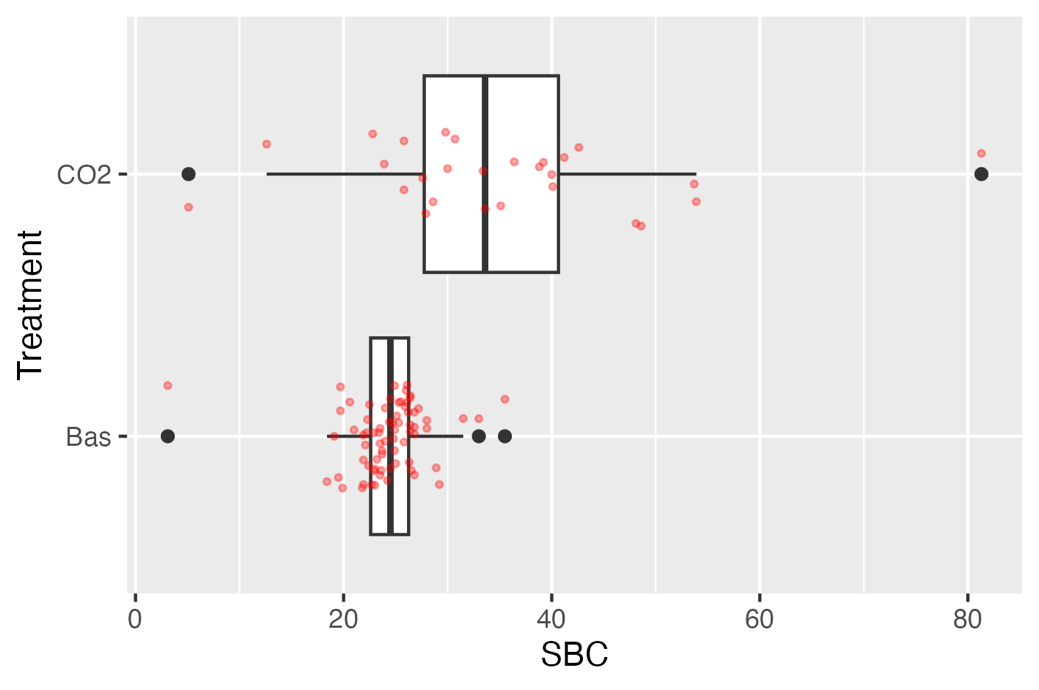


Fig. 15: Boxplots of variable SBC given treatment.


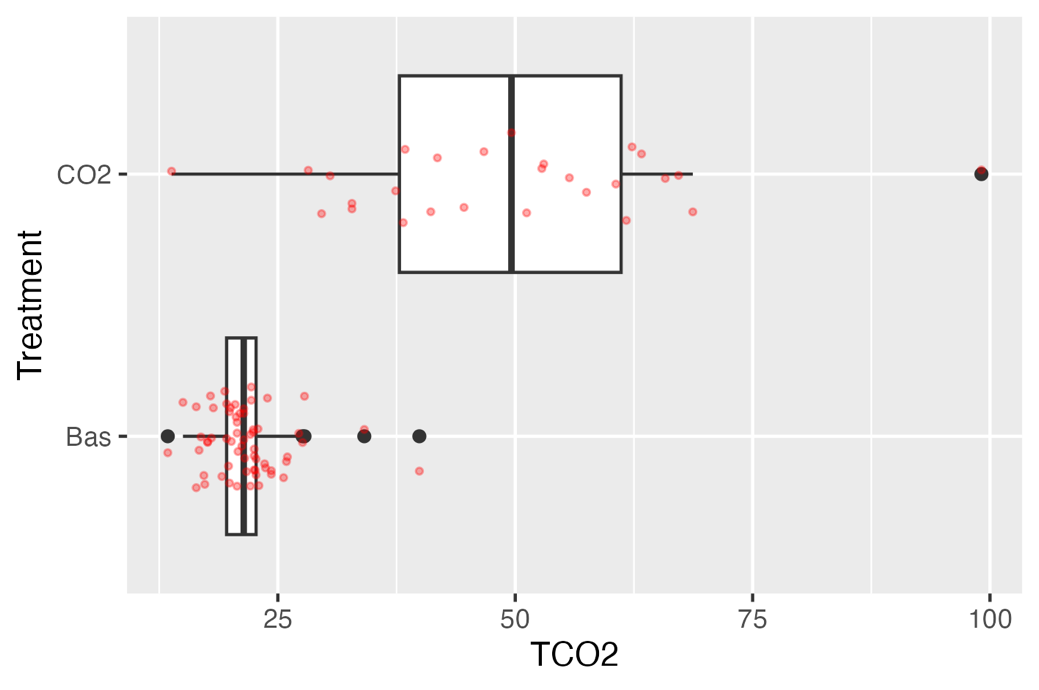


Fig. 16: Boxplots of variable TCO2 given treatment.
